# *In situ* evaluation of podocytes in patients with focal segmental glomerulosclerosis and minimal change disease

**DOI:** 10.1371/journal.pone.0241745

**Published:** 2020-11-04

**Authors:** Crislaine Aparecida da Silva, Maria Luíza Gonçalves dos Reis Monteiro, Liliane Silvano Araújo, Monise Gini Urzedo, Lenaldo Branco Rocha, Marlene Antônia dos Reis, Juliana Reis Machado

**Affiliations:** Department of Pathology, Genetics and Evolution, Discipline of General Pathology, Institute of Biological and Natural Sciences, Federal University of Triângulo Mineiro, Uberaba, Minas Gerais, Brazil; Center for Molecular Biotechnology, ITALY

## Abstract

Podocyte injury in focal segmental glomerulosclerosis (FSGS) and minimal change disease (MCD) results from the imbalance between adaptive responses that maintain homeostasis and cellular dysfunction that can culminate in cell death. Therefore, an *in situ* analysis was performed to detect morphological changes related to cell death and autophagy in renal biopsies from adult patients with podocytopathies. Forty-nine renal biopsies from patients with FSGS (n = 22) and MCD (n = 27) were selected. *In situ* expression of Wilms Tumor 1 protein (WT1), light chain microtubule 1-associated protein (LC3) and caspase-3 protein were evaluated by immunohistochemistry. The foot process effacement and morphological alterations related to podocyte cell death and autophagy were analyzed with transmission electronic microscopy. Reduction in the density of WT1-labeled podocytes was observed for FSGS and MCD cases as compared to controls. Foot process width (FPW) in control group was lower than in cases of podocytopathies. In FSGS group, FPW was significantly higher than in MCD group and correlated with proteinuria. A density of LC3-labeled podocytes and the number of autophagosomes in podocytes/ pedicels were higher in the MCD group than in the FSGS group. The number of autophagosomes correlated positively with the estimated glomerular filtration rate in cases of MCD. The density of caspase-3-labeled podocytes in FSGS and MCD was higher than control group, and a higher number of podocytes with an evidence of necrosis was detected in FSGS cases than in MCD and control cases. Podocytes from patients diagnosed with FSGS showed more morphological and functional alterations resulting from a larger number of lesions and reduced cell adaptation.

## Introduction

Focal segmental glomerulosclerosis (FSGS) and minimal change disease (MCD) are among the main causes of idiopathic nephrotic syndrome, termed as podocytopathies. These conditions show diffused foot processes effacement under electron microscopy [1].

Podocytes (visceral epithelial cells) are terminally differentiated cells lining the outer surface of glomerular capillaries, and their differentiation coincides with the progressive expression of maturity markers, including the Wilms tumor suppressor gene 1 (WT1) [2]. These cells appear to have an intrinsic system that could withstand tension but they may be undergo damage when the stresses exceed this capacity. Thus, podocyte injury is a result of the imbalance between the adaptive responses that maintain homeostasis and cellular dysfunction, leading to cell death [3].

To maintain homeostasis, podocytes have a high rate of autophagy, which is related to the formation of autophagosomes [4]. Proteins that are important for the formation of the autophagosome include light chain microtubule 1-associated protein (LC3), a member of the autophagy-related protein family 8 encoded by the *MAP1LC3* gene, which is required for the elongation and maturation of the autophagosome [5].

So, considering the research line of our group that has been looking for possible biomarkers, morphological alterations and mechanisms of lesions that may allow differentiation of the diagnosis of MCD from that of FSGS when sclerosis lesions are not sampled in renal biopsies [6–8], we propose to analyze *in situ* morphological and functional alterations related to apoptosis, necrosis and autophagy in renal biopsies of adult patients with FSGS and MCD.

## Materials and methods

This study was approved by the Ethics and Research Committee of the Federal University of Triângulo Mineiro with the number 2.949.713. All samples were archived and cases were identified by codes with letters and numbers to ensure that individuals were anonymized. Because it is a retrospective study, ethics committee waived the requirement for informed consent.

### Patients

A total of 49 cases of idiopathic podocytopathies in adults, including patients with FSGS (n = 22) and MCD (n = 27), were selected from the Nephropathology Service of the General Pathology Discipline of the Federal University of Triângulo Mineiro Uberaba, Minas Gerais, Brazil. FSGS group was defined by the presence of segmental and focal sclerosis (mesangial matrix increase) in light microscopy (LM) and foot process effacement in transmission electron microscopy (TEM). MCD group was defined by the absence of lesions in LM and foot process effacement as the only alteration in TEM analysis. All cases that presented morphological alterations compatible with other entities were excluded from this study. For analyzes by light microscopy, we used autopsy kidney fragments from patients whose death was not related to renal or infectious diseases to compose the control group (n = 18). Cases of non-glomerular proteinuria and isolated hematuria were selected to compose the control group (n = 12) for analyses by transmission electron microscopy.

### Renal histopathology

The kidney samples were evaluated by LM, immunofluorescence, TEM, and immunohistochemistry. For LM, the paraformaldehyde-fixed fragment was dehydrated in graduated alcohols, diaphanized in xylol, embedded in paraffin and submitted to serial cuts of 2μm thickness. The paraffin material slides were stained with hematoxylin and eosin, Sirius Red, periodic acid silver methenamine stain and Masson's trichrome. For direct immunofluorescence, the fragment was frozen in liquid nitrogen and sectioned with 2μm thickness sections. The frozen material slides were screened for IgA, IgG and IgM immunoglobulins, kappa and lambda light chains, C1q and C3 complement fractions, and fibrinogen by fluorescein isothiocyanate conjugated antibodies (Dako, Copenhagen, Denmark). For TEM, the sample was fixed with Karnovsky 2.5% + ruthenium red 0.2% and post-fixed in osmium tetroxide 2%, then dehydrated in graded alcohols and acetone solutions and embedded in Epon 812 resin. Ultrathin 60-nm-thick sections were cut and placed onto nickel grids and were subsequently contrasted with lead citrate 1% and uranyl acetate 3% and examined under an EM-900 transmission electron microscope (Zeiss, Germany) [8, 9].

### Immunohistochemistry for WT1, LC3, and caspase 3

Sequential histological sections of 2 μm of paraffin-embedded fragments were subjected to immunohistochemistry (Table 1) which was performed manually using the Novolink non-biotinylated polymer system (Novolink Polymer Detection System Kit, BL, UK).

**Table 1:** Immunohistochemistry specifications.

Primary antibody	Supplier	Clone	Antigen retrieval buffer	Antibody dilutions	Catalog numbers
**Monoclonal mouse anti-human Wilms’ tumor 1**	Dako	6F-H2	Citrate pH 6.0	1:500	M3561
**Monoclonal mouse anti-human LC3**	BIORBYT	166AT1234	Citrate pH 6.0	1:1200	orb97657
**Monoclonal rabbit anti-human Caspase 3**	NSJBIO	RM250	Citrate pH 6.0	1:1000	R20270

### Immunostaining quantification

All glomeruli without sclerosis of each sample were analyzed. Digital images of each glomerulus were captured using the AxionCam ICc5 (Zeiss, Germany) digital camera attached to the light microscope on the 40x objective lens. Immunostained cells showing an intense brownish staining and located outside glomerular loop were defined as podocytes. For each case, all immunostained brownish-colored podocytes were quantified in each glomerulus and the area of each glomerulus was measured. The result was expressed in cell density per glomerular area according to Venkatareddy et al. [10].

### Transmission electron microscopy

In TEM were evaluated the foot process width, the number of autophagosomes in podocytes / pedicels and evidence of necrosis in podocytes. Two glomeruli from each patient were evaluated and the images of all viable glomerular loops and all available podocytes were captured at an amplification of 7000X and the image analysis was performed with ImageJ 1.5 software.

To determine the foot process width (FPW), the length of the glomerular basement membrane (GBM) was measured and the number of foot process was counted similar to the Deegens et al. technique [11]. Each foot process was defined as a secondary epithelial segment connected to the GBM between two slit diaphragms. Thus, the FPW was obtained by dividing the number of foot process counted by the length of GBM analyzed [11] corrected by the correction factor π / 4 to exclude the assumed random variation in the section angle in relation to the long axis of the podocyte [12]. For each patient, the average foot process width was expressed in nanometers.

To evaluate podocyte number, all available podocytes were captured at an amplification of 7000X and their nucleus were observed in each image, without repeating fields. So, for each case the average number of podocytes was obtained.

The number of autophagosomes in podocytes/foot process and the number of podocytes with evidence of necrosis were counted in each image without repeating fields and the results were expressed as the average number of each variable analyzed for each case. Autophagosomes were identified as vesicles containing undigested cytoplasmic material [13]. Evidence of necrosis in podocytes were oncosis (increased cell volume), nuclear edema (increased nuclear volume with decreased electrodensity) and cell lysis (cytoplasmic membrane rupture and loss of intracytoplasmic content) [14].

### Statistical analysis

Statistical analysis was performed using the GraphPad Prism software version 7.0. The normality in the frequentist statistics was tested by the Shapiro Wilk test. Comparison between cases with normal distribution and similar variations, the ANOVA (F) test was used, followed by the Tukey post-test or Student's t test (t). The Kruskal-Wallis tests (H), followed by the Dunn post-test or Mann-Whitney test (U) were used to compare cases with non-normal distribution. For the analysis of associations, the chi-square test (χ2) was used. Correlations were assessed with the Pearson (r) and Spearman (rS) tests. Differences were considered significant when p <0.05.

## Results

### Epidemiological and clinical-laboratory analysis of patients

Of the 49 cases of podocytopathies studied in adults, the majority were male (53.06%) and white (71.43%), with a mean age of 35.4 ± 13.0 years. Among patients with FSGS, 13 (59.1%) were male and 15 (68.2%) were white, and the mean age was 35.1 ± 2.5 years. Of all patients with MCD, 13 (63.6%) were male and 20 (74.1%) were white, while their mean age was 35.6 ± 13.4 years. In the control group, 9 (50.0%) patients were males and 9 (50.0%) were females, and the mean age was 42.8 ± 13.2 years.

Patients from both groups had nephrotic proteinuria, with a mean value of 6.3 ± 4.9 g/24 h in FSGS group and 4.4 ± 4.2 g/24 h in MCD group. The mean serum albumin level was 2.6 ± 0.9 mg/dL in patients with FSGS and 2.7 ± 0.8 mg/dL in those with MCD. The mean serum creatinine level was 1.5 *±* 1.0 mg/dL and 1.0 *±* 0.5 mg/dL and the average estimated glomerular filtration rate (eGFR) was 72.9 *±* 39.9 mL/min/1.73m^2^ and 88.4 *±* 28.4 mL/min/1.73m^2^ in FSGS and MCD groups, respectively. Hematuria was reported in 6 (27.3%) patients with FSGS group and 10 (37.0%) patients with MCD, and systemic arterial hypertension was present in 16 (72.7%) patients in the FSGS group and in 7 (25.9%) patients in the MCD group.

### Morphological analysis of patient biopsies

At least 10 glomeruli per patient were evaluated in LM and in TEM analysis, at least two glomeruli were evaluated per patient and an average of 12.8 ± 5.4 podocytes and 15.8 ± 5.6 glomerular loops were observed with a length of 12552.6 ± 1314.8 nm (Table 2).

**Table 2:** Morphological analysis.

	**Control (n = 18)**	**FSGS (n = 22)**	**MCD (n = 27)**
**Number of WT1-labeled podocytes**			
*Mean ± SD*	28.3±4.7	18.0±7.8	18.6±6.4
*Median (Min-Max)*	28.0 (19.0–38.0)	17.0 (7.0–37.0)	17.5 (9.0–34.0)
	**Control (n = 12)**	**FSGS (n = 22)**	**MCD (n = 27)**
**Number of evaluated podocytes in TEM**			
*Mean ± SD*	11.0±5.5	11.1±4.5	13.6±5.7
*Median (Min-Max)*	9.5 (4.0–21.0)	10.5 (5.0–19.0)	12.0 (4.0–25.0)
**Number of glomerular loops in TEM**			
*Mean ± SD*	15.5±6.3	15.0±5.2	16.2±5.9
*Median (Min-Max)*	15.0 (7.0–28.0)	15.0 (6.0–23.0)	16.0 (5.0–25.0)
**Length of glomerular loops in TEM (nm)**			
*Mean ± SD*	11962.4±1370.9	12944.4±998.4	12280.0±1458.3
*Median (Min-Max)*	11570.6 (10149.3–15151.0)	13000.0 (11000.0–14000.0)	12000.0 (11000.0–18000.0)
**Foot process widht (nm)**			
*Mean ± SD*	471.0±101.9	2503.0±1506.5	1488.9±793.4
*Median (Min-Max)*	452.1 (395.2-777-3)	2739.2 (493.0–5550.3)	1327.6 (521.2–3011.8)

FSGS, Focal segmental glomerulosclerosis; n, Number of cases; WT1, Antibody anti-human Wilms’tumor 1 protein; SD, Standard deviation; Min, Minimum; Max, Maximum; TEM, Transmission electronic microscopy; nm, Nanometer

**Fig 1:**
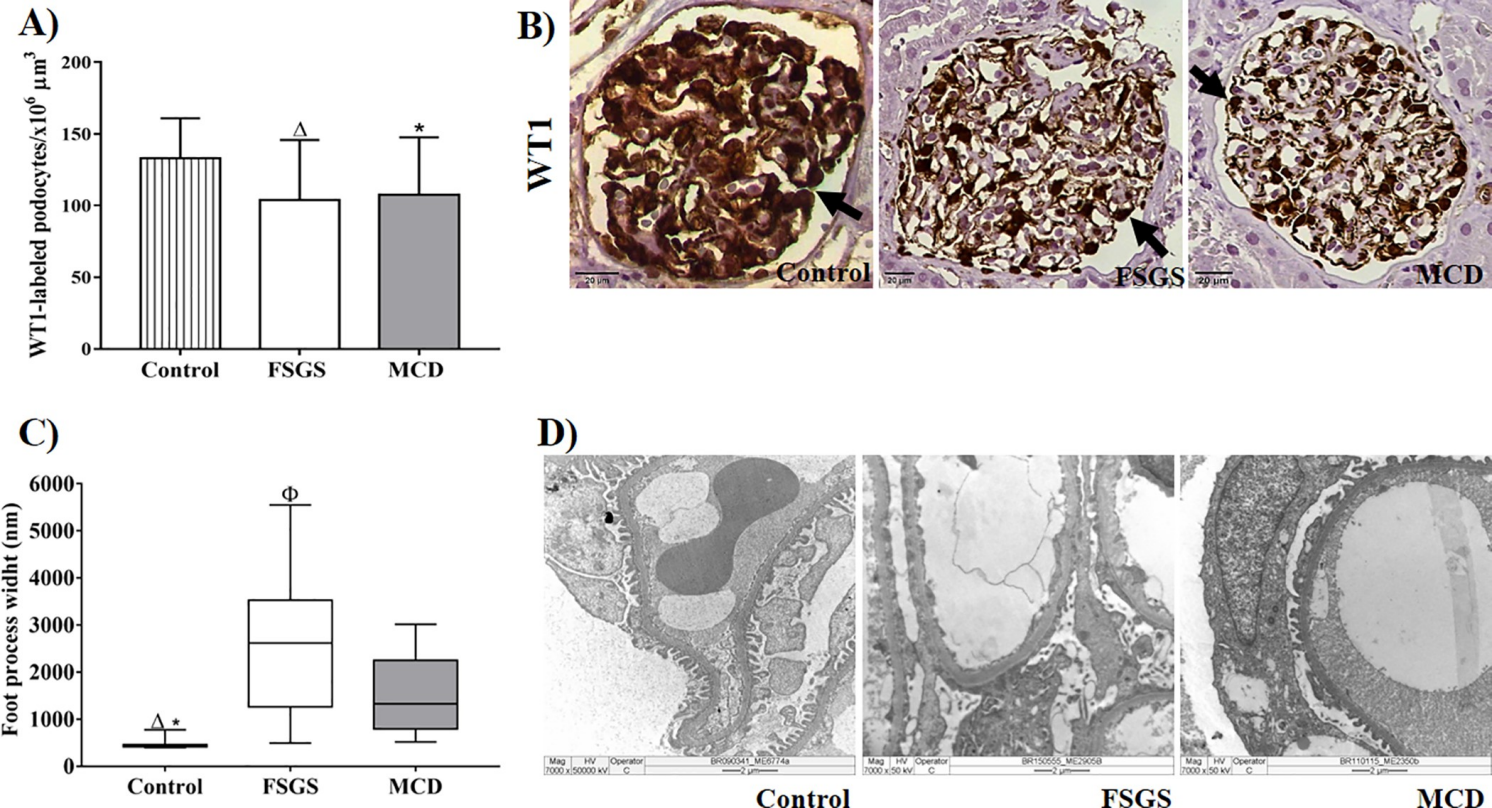
Morphological evaluation of podocytes in renal biopsies of patients with FSGS and MCD. (A) Density of WT1-labeled podocytes in three groups. ANOVA test followed by Tukey’s multiple comparison test. The bars represent the mean and the vertical lines represent the standard error of the mean. (B) WT1-labeled podocytes in the control, FSGS, and MCD groups (arrows). (C) Difference in the foot process width between control, FSGS and MCD groups showing the extensive foot process effacement in FSGS group. Kruskal-Wallis test followed by Dunn's multiple comparisons test. The horizontal lines represent the medians, bars represent the 25–75% percentiles, and the vertical lines represent the 10–90% percentiles. (D) TEM showing more foot process effacement in a case of FSGS and more foot process preserved in a case of MCD and in a case of control group. Δ: Significant difference between FSGS and control group. *: Significant difference between MCD and control group. Φ: Significant difference between FSGS and MCD group.

Previous results from our group have shown that podocytes have increased expression of urokinase plasminogen activator receptor (uPAR) *in situ* in biopsies from adult [8] and pediatric [9] patients with morphological lesions compatible with FSGS. From these findings, we evaluated the possible causes of cellular injury in podocytopathies. As these diseases progress with changes in podocytes, we evaluated the number of podocytes using the expression of WT1 and observed a significant reduction in the density of podocytes both in FSGS and MCD cases as compared to controls (Fig 1A and 1B, p = 0.0430, F = 0.1014, Table 2). We also evaluated the functionality of podocytes by measuring their width. We observed that FPW was higher in the cases of FSGS and MCD than in control group. Among podocytopathies, FPW was greater in FSGS group than in MCD [(Fig 1C and 1D, p<0.0001, H = 29.09), Table 3].

**Table 3:** Podocytes lesions and adaptations analysis in renal biopsies of patients with FSGS and MCD.

	**Control (n = 18)**	**FSGS (n = 22)**	**MCD (n = 27)**
***Autophagy evidences***			
**Number of LC3-labeled podocytes**			
*Mean ± SD*	25.6±12.4	14.5±4.0	15.8±5.5
*Median (Min-Max)*	20.0 (15.0–52.0)	14.0 (10.0–24.0)	16.0 (9.0–27.0)
	**Control (n = 12)**	**FSGS (n = 22)**	**MCD (n = 27)**
**Number of autophagosomes in TEM**			
*Mean ± SD*	139.2*±*60.9	54.0±25.6	79.2±40.3
*Median (Min-Max)*	134.0 (47.0–235.0)	55.0 (10.0–92.0)	80.0 (20.0–150.0)
**Number of autophagic vesicles in TEM**			
*Mean ± SD*	4.0*±*2.4	2.9±2.7	4.4±3.4
*Median (Min-Max)*	4.0 (0.0–8.0)	2.0 (0.0–10.0)	4.0 (0.0–11.0)
***Apoptosis evidences***			
**Number of Caspase 3-labeled podocytes**			
*Mean ± SD*	2.4±0.9	7.6±2.9	6.9±1.4
*Median (Min-Max)*	2.0 (1.0–4.0)	8.0 (3.0–12.0)	7.0 (5.0–10.0)
***Necrosis evidences***			
**Number of podocytes with oncosis in TEM**			
*Mean ± SD*	0.8*±*1.2	0.3±0.6	0.6±1.0
*Median (Min-Max)*	0.0 (0.0–3.0)	0.0 (0.0–2.0)	0.0 (0.0–4.0)
**Number of podocytes with nuclear edema in TEM**			
*Mean ± SD*	0.0±0.0	0.3±0.8	0.1±0.3
*Median (Min-Max)*	0.0 (0.0–0.0)	0.0 (0.0–3.0)	0.0 (0.0–1.0)
**Number of podocytes with cell lysis in TEM**			
*Mean ± SD*	0.0±0.0	0.3±1.0	0.0±0.0
*Median (Min-Max)*	0.0 (0.0–0.0)	0.0 (0.0–4.0)	0.0 (0.0–0.0)

FSGS, Focal segmental glomerulosclerosis; MCD, Minimal change disease; n, Number of cases; LC3, Antibody anti-human LC3; SD, Standard deviation; Min, Minimum; Max, Maximum; TEM, Transmission electronic microscopy; Caspase 3, Antibody anti-human Caspase 3

### Podocytes of FSGS patients show more lesions and less cellular adaptation than those of MCD patients

**Fig 2:**
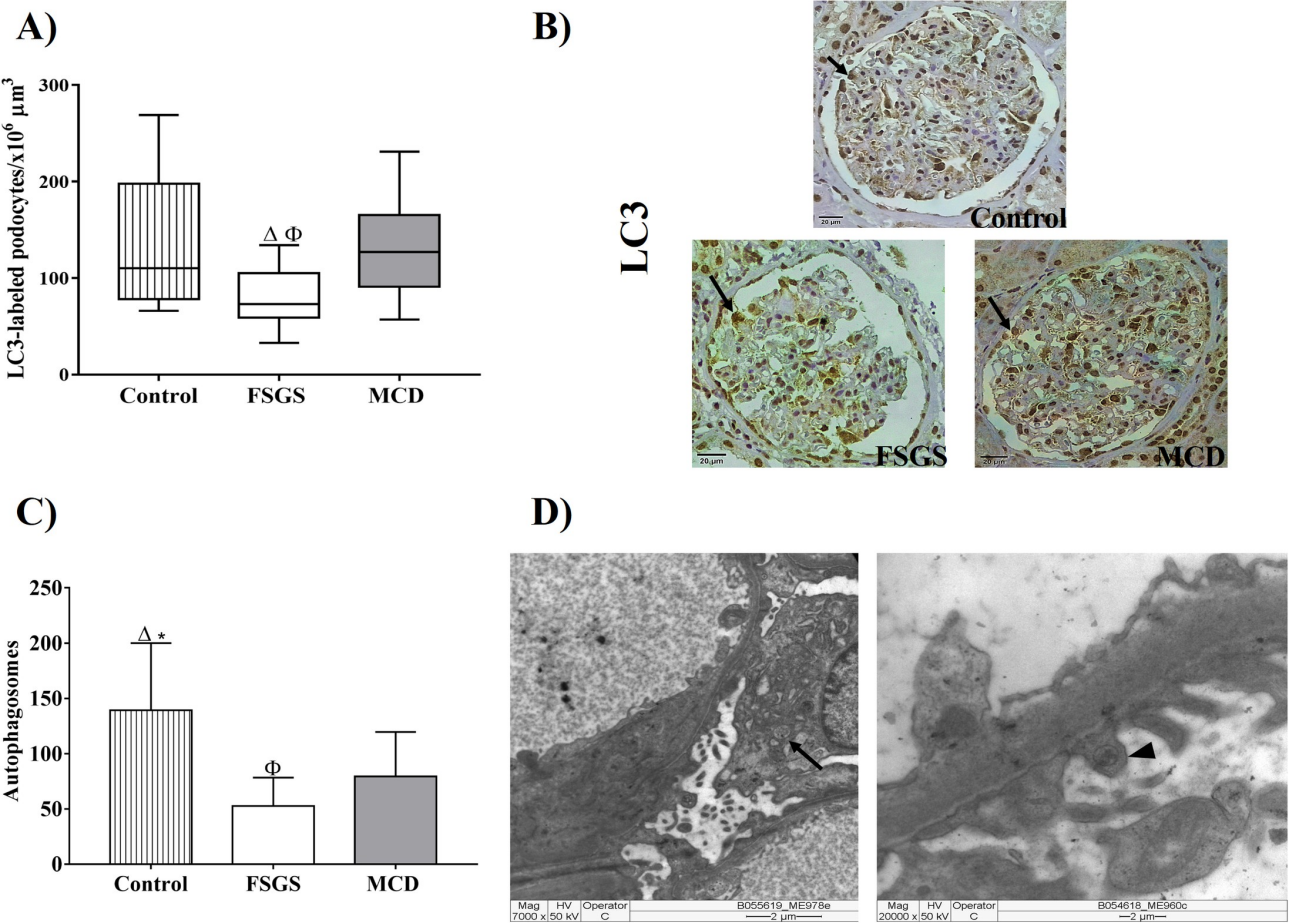
Evidence of podocyte autophagy in renal biopsy of patients with FSGS and MCD. (A) Density of LC3-labeled podocytes in three groups. ANOVA test followed by Tukey’s multiple comparison test. The bars represent the mean and the vertical lines represent the standard error of the mean. (B) LC3-labeled podocytes in the control, FSGS, and MCD groups (arrows). (C) Difference in the number of autophagosomes between control, FSGS and MCD groups implies higher autophagy in MCD and control group. ANOVA test followed by Tukey’s multiple comparison test. Bars represent the mean and vertical lines represent standard error of the mean. (D) TEM showing autophagosomes characterized as a multivesicle bodies containing undigested cytoplasmic material in podocytes (arrows) and pedicels (arrow head). Δ: Significant difference between FSGS and control group. Φ: Significant difference between FSGS and MCD group.

As FSGS group showed a functional reduction in podocytes, we used LM and TEM to find evidences of lesions and cellular adaptations in podocytes. Glomeruli of FSGS patients showed lower density of LC3-labeled podocytes, evidence of autophagy when compared to the control group and the MCD group (Fig 2A and 2B, p = 0.0162, H = 8.243, Table 3). A greater number of autophagosomes was observed in podocytes and pedicels of patients from control group compared with FSGS and MCD groups. Besides, podocytes and pedicels of patients with MCD showed great number of autophagosomes than in those with FSGS (Fig 2C and 2D, p<0.0001, F = 16.15, Table 3), showing that autophagy is less prevalent in podocytes of FSGS cases.

**Fig 3:**
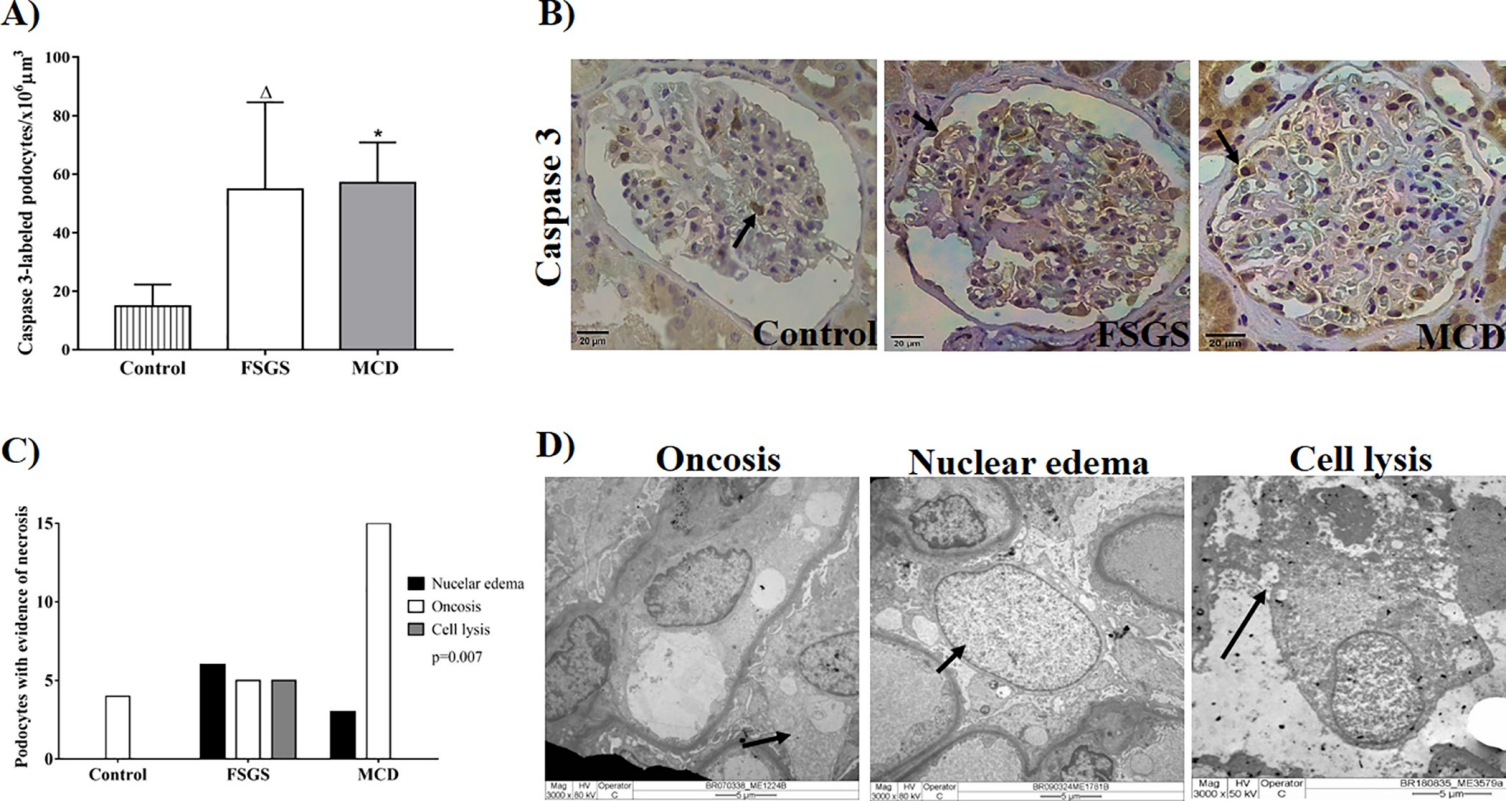
Evidence of cell death in renal biopsy of patients with FSGS and MCD. (A) Density of podocytes labeled with caspase-3 in three groups. ANOVA test followed by Tukey’s multiple comparison test. The bars represent the mean and the vertical lines represent the standard error of the mean. (B) Caspase 3-labeled podocytes in the control, FSGS, and MCD groups (arrows). (C) Morphological evidence of necrosis in podocytes from control, FSGS and MCD group, indicating a higher number of podocytes with nuclear edema and cell lysis in FSGS cases. Chi-square test. (D) TEM illustrating podocytes (arrows) with morphological alterations compatible with necrosis. Δ: Significant difference between FSGS and control group. *: Significant difference between MCD and control group.

After the analysis of podocyte viability through autophagy, the possible mechanisms underlying cell death in podocytes were evaluated. A higher density of caspase-3-labeled podocytes were observed in glomeruli of patients from the FSGS group and from the MCD group compared to the control group (Fig 3A and 3B, p < 0.0001, F = 23.69, Table 3). Thus, apoptosis-mediated death of podocytes occurs in both podocytopathies. However, a higher number of podocytes with evidence of necrosis such as nuclear edema and cell lysis was observed in FSGS cases than in MCD and control cases (Fig 3C and 3D, p = 0.007, χ2 = 14.09, Table 3). Cell lysis, a morphological characteristic of necrosis, was absent in all MCD and control cases.

### Evaluation of impaired podocyte function

**Fig 4:**
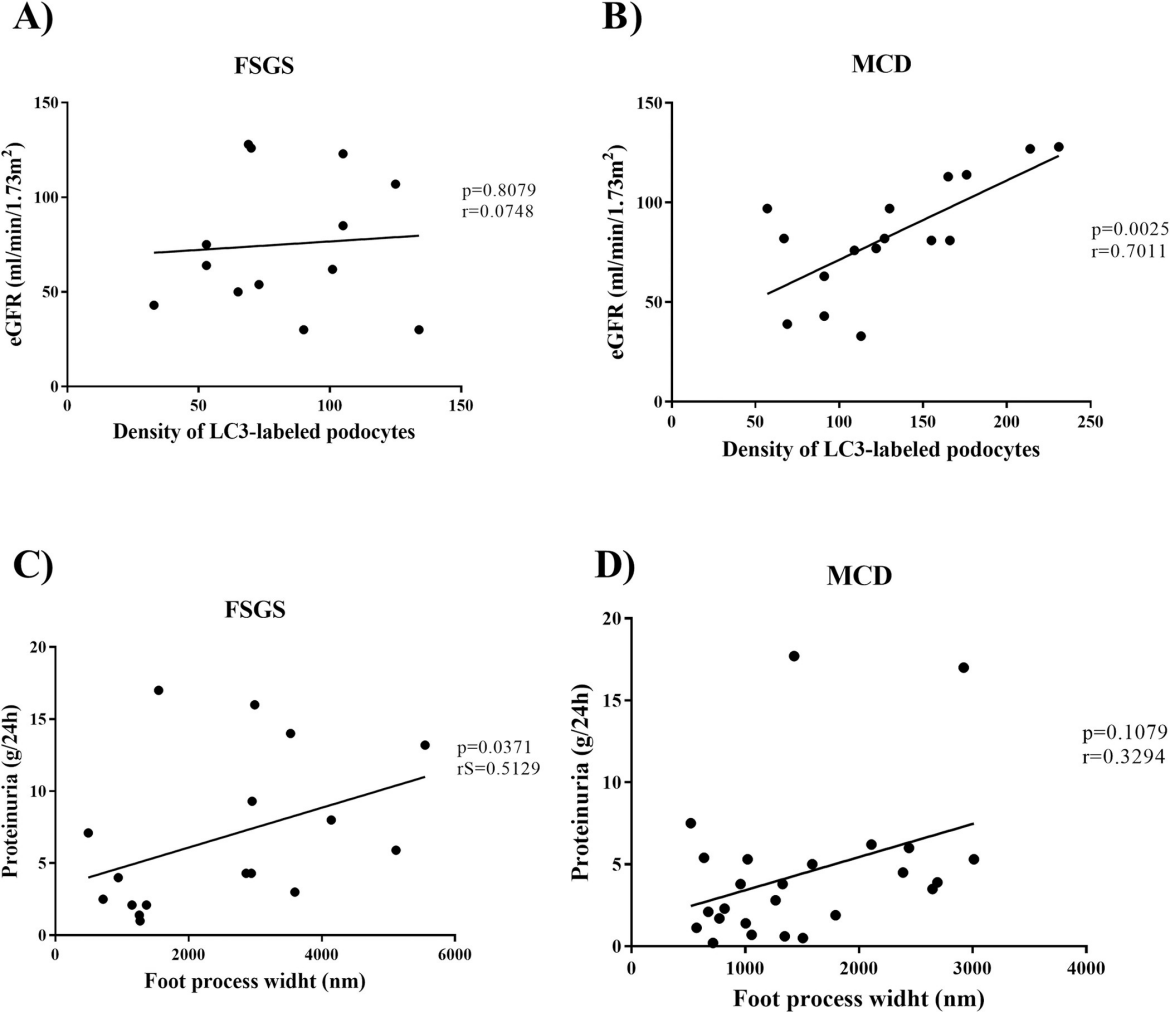
Evaluation of impaired podocyte function. Correlation between the estimated glomerular filtration rate and density of LC3-labeled podocytes in (A) FSGS and (B) MCD group. Correlation between proteinuria and foot process width in (C) FSGS and (D) MCD group. r: Pearson's correlation coefficient. rS: Spearman’s correlation coefficient.

As podocytes from FSGS group showed morphological changes and those from MCD group exhibited better cell adaptation properties, we evaluated the correlation between morphological alterations and relevant clinical data in patients with podocytopathies. A significant positive correlation was detected between eGFR and density of LC3-labeled podocytes in MCD cases (Fig 4B, p = 0.0025, r = 0.7011), which was not observed in FSGS cases (Fig 4A, p = 0.8079, r = 0.0748). Patients with FSGS showed positive and significant correlation between proteinuria levels and FPW (Fig 4C, p = 0.0371, rS = 0.5129) that was absent in patients from MCD group (Fig 4C, p = 0.1079, r = 0.3294).

This result indicates that proteinuria exhibits a direct relationship with the extension of foot process effacement in FSGS group.

To complement and validate our results, we performed a comparative analysis between sclerotic and non-sclerotic glomeruli. Analyzing all glomeruli available in each sample (with and without sclerosis), a significant reduction in the density of WT1-labeled podocytes both in FSGS and MCD cases was observed compared to control group (p = 0.0390; F = 3.44). Glomeruli of FSGS patients showed lower density of LC3-labeled podocytes compared to the control group and MCD group (p = 0.0297; F = 3.801). A higher density of caspase-3-labeled podocytes were observed in glomeruli of patients from FSGS group and from MCD group compared to control group (p < 0.0001, F = 23.75). Thus, the comparison between sclerotic and non-sclerotic glomeruli showed results similar to those previously reported in the study.

## Discussion

The podocytopathies FSGS and MCD are glomerular diseases that are among the main causes of nephrotic syndrome. A podocyte is a primarily damaged cell and the extent of damage may vary [1].

One way to evaluate podocyte loss is through the analysis of specific podocyte markers such as WT1, which is related to the maintenance of podocyte differentiation [2]. We investigated whether there exists any differences in the density of podocytes in renal biopsies of patients with FSGS and MCD through immunostaining for WT1. As a result, we found a significant reduction in the density of WT1-labeled podocytes both in FSGS and MCD cases as compared to controls, but there was no difference between the two podocytopathies. Animal model studies have demonstrated the relationship between reduction in WT1-labeled podocytes and glomerulosclerosis [15]. However, we have previously shown that there is no difference in the density of WT1-labeled podocytes in biopsies of pediatric patients with FSGS and MCD [9]. Unlike other studies, we excluded glomeruli that presented sclerosis. We believe a possible explanation would be that mechanisms of podocyte injury may cause phenotypic podocyte modifications, resulting in reduced expression of markers in situ, such as WT1, in both podocytopathies [16]. In addition, another mechanism would be that parietal epithelial cells (PECs), which are progenitors of podocytes, invade glomerular tuff and replace podocytes. This way, PECs can play a protective role in glomerular diseases, such as FSGS, responding to damage to podocytes with proliferation [17, 18]. Another possibility is that podocytes detach from the glomerular loop in response to hypertrophic stress analogous to the “mitotic catastrophe” model suggested by Lasagni et al. [19] and similar to what is observed in aging human glomeruli [20] which causes a reduction in WT1 immunostaining. Although we have not been able to identify detached podocytes in Bowman's space or tubular lumens by histology, and the number of podocytes per glomerulus was not measurable in association with FSGS development, it is possible that the methods used were not sensitive enough to detect these events in a subset of glomeruli.

The common ultrastructural findings between FSGS and MCD is the foot process effacement resulting from change in the binding complex between GBM and the pedicel, leading to a disorder in actin cytoskeleton [21]. To measure podocyte lesion, the extension of foot process effacement was assessed by the evaluation of mean FPW which was higher in FSGS cases than in MCD cases. Thus, a more diffused foot process effacement was noted in patients with FSGS. This observation has been demonstrated in a quantitative study, wherein the mean of FPW was higher in FSGS cases than in MCD cases [11].

The difference in foot process effacement between FSGS and MCD cases, suggests that podocyte damage in these diseases occurs owing to different mechanisms, which is reinforced by the difference in the expression of some podocyte proteins [6, 7]. It has been reported that the expression of dystroglycan, an adhesion molecule between the podocyte and GBM, is significantly lower in biopsies of patients with MCD than in those of patients with FSGS [22]. Another protein, uPAR, is upregulated in podocytes from cases as compared to those from MCD cases [8, 9] and is related to the activation of β3 integrin, leading to the foot process effacement in FSGS [23]. In this study, the mean FPW showed a positive and significant correlation with the levels of proteinuria in FSGS cases. The mean FPW also positively correlated with proteinuria in cases of proteinuric diseases, including FSGS [24] and IgA nephropathy [25]. However, the relation between foot process effacement and proteinuria is still controversial. Some morphometric studies that evaluated the FPW and its correlation with proteinuria failed to show this relationship, suggesting that proteinuria mainly depends on the nature of the underlying disease and not on the effacement [11, 26]. In experimental models with puromycin aminonucleoside nephrosis, effacement precedes proteinuria [27]. In addition, it has been shown that effacement is associated with narrowing of glomerular filtration slits and development of tight junctions between foot processes [28]. These data suggest that, after foot process effacement by some specific cause, this leads to proteinuria. Therefore, we suggest in our study that, possibly, proteinuria may be associated with impaired function of podocytes, as this clinical sign may reflect the impairment of the selective permeability function of podocytes that favors protein leakage to the urinary space, indicating decreased glomerular function. So, in agreement with another study, we believe that once effacement is initiated, the degree of proteinuria correlates with the degree of effacement [25].

In general, podocyte lesion evolves with better prognosis in MCD but progresses more easily to renal disease at terminal stages in FSGS [29]. Accordingly, we assessed whether podocytes from patients with MCD could better adapt to injury than those from patients with FSGS. We investigated the mechanism underlying podocyte autophagy, a process that maintains cellular homeostasis [30]. We observed that MCD cases showed higher density of LC3-labeled podocytes and higher of autophagosomes than FSGS cases, indicating that podocytes from MCD could adapt better than those from patients with FSGS. Furthermore, we observed a positive and significant correlation between the density of LC3-labeled podocytes and eGFR. Ogawa-Akiyama el al, demonstrated positive areas of LC3 co-located with positive areas of WT1 in immunofluorescence, suggesting the occurrence of autophagy in podocytes of patients with MCD [31]. A study with renal biopsies of patients with MCD and with FSGS showed that podocytes from MCD patients had higher levels of Beclin-mediated autophagic activity1 than podocytes of FSGS patients. In addition, repeated renal biopsies from patients with MCD made it possible to track autophagic activity of podocytes and confirmed patients that maintained high autophagic activity of podocytes maintained the status of MCD, while patients with podocytes with decreased autophagic activity, evolved to FSGS, demonstrating that the autophagic activity of podocytes plays a critical protective role in kidney injury in cases of podocytopathies [32]. So, our findings allow the suggestion that autophagy may be playing a protective role against podocyte lesions in cases of MCD.

Podocyte dysfunction may lead to death due to apoptosis and necrosis [33]. Morphological manifestations of cell death have historically been employed to classify cell death in three different forms. Type I cell death corresponds to apoptosis and is characterized with little or no ultra-structural modifications in cytoplasmic organelles, formation of apoptotic blebs and bodies, maintenance of the integrity of the plasmatic membrane until the final stage, and phagocytosis by resident phagocytes. Type II cell death is defined as an autophagy-dependent cell death with the formation of large-scale autophagic vacuolization, which may cause cell death under specific circumstances; however, autophagy may promote cell survival. Type III cell death or necrosis is morphologically characterized by increased cell volume (oncosis), organelle swelling, rupture of the plasma membrane, and subsequent loss of intracellular content [14].

Podocyte apoptosis has been proposed as a mechanism of podocyte loss and glomerulosclerosis in a transforming growth factor -β1 transgenic mouse model [34], a *knockout* for CD2AP [35], a puromycin-induced lesion model [15], and mouse and human glomerular epithelial cell cultures [36]. In human samples, *in situ* apoptosis was observed only in cases of lupus nephritis [37] and IgA nephropathy [38]. In the present study, no difference was observed in the analysis of apoptosis with caspase-3 immunostaining, and this finding differs from the literature that reports the existence of a relationship between apoptosis and glomerulosclerosis. However, apoptosis reported in the literature was observed at a higher frequency in cultured podocytes than in podocytes *in vivo* which demonstrates a technical difficulty in analyzing apoptosis *in situ*. Besides caspase 3, caspase 6 and 7 are also apoptotic effector caspases. Thus, another hypothesis that did not find difference in apoptosis among the studied podocytopathies is that this form of cell death may occur through a caspase-3-independent pathway [39, 40].

On the other hand, higher number of podocytes with evidence of necrosis was reported in FSGS cases, proving that these cases, besides presenting fewer cell adaptations, have more lesions. Necrosis as a mechanism of podocyte cell death is less studied, probably owing to technical limitations and difficulties in detecting necrotic structures *in vivo*. An example of podocyte necrosis was demonstrated in a transplanted kidney biopsy sample from a patient with severe acute renal failure secondary to antibody-mediated rejection, wherein a podocyte with oncosis and cell lysis was observed [41]. Most necrotic podocytes probably detach from the GBM early in the process and are lost in the tubular system [33].

In our study, podocytes from patients with FSGS showed more evidence of cell death and less autophagy than podocytes from patients with MCD, suggesting that in FSGS cases podocytes present more lesions and fewer cell adaptations.

## Conclusions

Podocytes of patients with FSGS present fewer cellular adaptations and more morphological and functional changes and that these changes may be related to the mechanisms of cell death by necrosis and caspase 3-independent apoptosis.
